# Added effect of 1% topical alendronate in intra-bony and inter-radicular defects as part of step II periodontal therapy: a systematic review with meta-analysis and trial sequential analysis

**DOI:** 10.1186/s12903-022-02044-1

**Published:** 2022-01-21

**Authors:** Claudia Arena, Vito Carlo Alberto Caponio, Khrystyna Zhurakivska, Lucio Lo Russo, Lorenzo Lo Muzio, Giuseppe Troiano

**Affiliations:** Department of Clinical and Experimental Medicine, University of Foggia, 71122 Foggia, Italy

**Keywords:** Periodontal defects, Periodontitis, Non-surgical treatment, Alendronate, Meta-analysis, Systematic review

## Abstract

**Background:**

This systematic review and meta-analysis aimed to investigate the role of alendronate combined with step 2 of periodontal therapy in reducing probing pocket depth, improving clinical attachment level, and reducing bone defect depth in intra-bony and inter-radicular defects.

**Methods:**

RCTs with more than 6 months follow-up were included in this study. Risk of bias assessment was performed using the Cochrane collaboration tool. In addition, meta-analysis and trial sequential analysis were used to aggregate the available evidence.

**Results:**

Seven studies met the inclusion criteria and were included in the systematic review. Topical application of alendronate during second step of periodontal therapy significantly improved PD and CAL.

**Conclusion:**

Local application of alendronate may confer a beneficial effect when applied during step II of periodontal therapy even if long term studies are needed to confirm these results.

**Clinical relevance:**

Considering the emerging role of host-inflammatory response in treatment of periodontitis and the antiresorptive and osteostimulative properties of bisphosphonates, several studies are focusing on the role of alendronate as an addition to non-surgical periodontal therapy.

**Supplementary Information:**

The online version contains supplementary material available at 10.1186/s12903-022-02044-1.

## Background

Periodontitis is an inflammatory disease affecting tissues surrounding teeth, characterized by destruction of connective tissue attachment and alveolar bone [1]. It is mainly caused by the bacterial biofilm which is responsible for the inflammatory and immunologic reaction that leads to the loss of connective tissue attachment and alveolar bone [2]. Therefore, second step of periodontal therapy is considered crucial to remove the bacterial biofilm both in its soft and calculus form in order to reestablish periodontal health in all patients with loss of periodontal support and/or periodontal pocket formation [3]. This is usually followed by home care measures and in the end by surgery. But while the complete disintegration of the bacterial biofilm can lead to the removal of the cause of the inflammation, several studies demonstrated the role of host inflammatory response in the breakdown of connective tissue and bone and therefore for disease progression [4]. Recent studies explored the use of host-modulators to reduce periodontal disease by altering the inflammatory response [5]. Bisphosphonates (BPs) are carbon-substituted pyrophosphate analogs that bind the mineral component of the bone interfering with the action of osteoclasts [6]. They find application in post-menopausal women for prevention and treatment of osteoporosis [7], in bone related diseases like Paget’s disease and hypercalcemia of malignancy [8]. Some studies showed that BPs induced osteoclasts to secrete inhibitors of osteoclast mediated resorption and stimulated the formation of osteoblast precursors and mineralized nodules, thus promoting bone formation [8, 9]. The role of BPs in treatment of periodontitis has been analyzed in an animal study in order to detect its potential role in retarding bone loss around teeth affected by periodontitis [10]. BPs showed a potential role against bone loss when systemically administered; moreover local adjunct to scaling and root-planning (SRP) caused a decreasing in bone loss and improving mineral density [11]. However, among different BPs, local administration of high doses of alendronate in periodontal pockets, could stimulate the release of IL-1 and IL-6 thus increasing host inflammatory response [12, 13]. Alendronate is an amino bisphosphonate commonly used as a potent inhibitor of bone resorption. However, to be effective, it needs to be administered in high dosage to maintain the necessary concentration of the drug at the osseous defect and systemic administration of BPs can cause several side effects to the gastro-intestinal tract, renal failure and severe hypocalcemia [14]. For these reasons local application might be more successful in controlling local concentration and reducing toxicities. Previous studies demonstrated its role in decreasing bone loss and increasing the bone density of alveolar bone [6]. The aim of this study was to systematically review the role of local 1% alendronate gel in non-surgical therapy of intra-bony and inter-radicular defects.

## Methods

### Protocol, registration and focused question

A systematic review protocol was written in the planning stages and both the Cochrane Handbook and the PRISMA (“Preferred Reporting Items for Systematic Reviews and Meta-Analyses”) statement were followed for the planning and reporting of the review. In addition, the protocol of this systematic review was registered on the PROSPERO database (registration code: CRD42021223883).

This review was performed aiming to answer the following PICO question: “Do intra-bony and inter-radicular defects (Participants) heal better with the adjunct of 1% alendronate (Intervention) to SRP instead of placebo (Comparison) in terms of PD, CAL and bone defect depth (Outcomes)?

#### Inclusion and exclusion criteria

Only studies fulfilling the following inclusion criteria were considered eligible for inclusion in this review: (1) *Type of studies*: Randomized controlled trials with a follow-up of at least 6 months. A shorter follow up was not considered as it would be unlike to reflect a meaningful difference in treatment response between test and control; (2) *Types of participants*: Adults (> = 18 years old), systematically healthy individuals diagnosed with periodontitis; (3) *Types of intervention*: Studies evaluating the adjunctive use of alendronate 1% gel administered locally during step 2 of periodontal therapy in intra-bony and inter-radicular defects; *Comparison*: patients receiving placebo or none adjunct treatment during step II of periodontal therapy; (4) *Outcomes:* Primary outcome: reduction in PD, CAL gain and bone defect depth. Hence, the following exclusion criteria were considered: (1) Non RCT or RCTs with a follow up < 6 months were excluded; (2) Studies involving less than 20 sites per group; (3) studies including participants with systemic diseases or that were taking medications were excluded.

### Information sources and search strategy

Studies were identified through an online search on PubMed, Scopus, and Web of Science. The search strategy included terms related to the population and the intervention. A combination of MESH terms and Free Text words combined with Boolean operators; for example in PubMed the following string was used: (((‘bisphosphonate’ OR ‘BP’ OR ‘alendronate’ OR ‘alendronate gel’) AND (‘osseous defects’ OR ‘intrabony defects’ OR ‘infrabony defects’ OR ‘furcation defects’ OR ‘furcation’ OR ‘periodontitis’))). A manual search was performed through several scientific journals, namely: Journal of Dental Research, Journal of Clinical Periodontology, Journal of Periodontology, International Journal of Periodontics and Restorative Dentistry, Journal of Oral and Maxillofacial Surgery. The bibliographies of pertinent review articles and studies finally included for data extraction were also screened.

### Study selection and data collection process

Eligibility of studies was assessed by two independent authors in a standardized manner (CA, VCAC). In the first round, records were screened by only reading title and abstract of publications. The studies assessed as eligible were included in the second round and underwent full-text reading. Only studies fulfilling the inclusion criteria were considered eligible and included in the review for the subsequent data extraction. Disagreements between authors were solved through discussion and a third author (GT) evaluated the agreement between reviewers by calculating a value of K-statistic. In addition, data extraction and collection were performed by two authors (CA, KZ) in a joint session using an ad hoc extraction sheet.

### Risk of bias assessment

The Cochrane Collaboration Tool was used for risk of bias assessment in the included studies [15]. The analysis was performed by two reviewers (CA and VCAC) in a joint session on the basis of seven domains: (a) random sequence generation, (b) allocation concealment, (c) selective reporting, (d) blinding of participants, (e) blinding outcome assessment, (f) incomplete outcome data and (g) other sources of bias. The judgment for each entry involved answering a question: the answer ‘YES’ meant low risk of bias, ‘NO’ meant high risk of bias and ‘UNCLEAR’ indicated either lack of information or uncertainty about potential biases. The GRADE methodology was used to assess the quality of the body of retrieved evidence (GRADEpro, Version 20. McMaster University, 2014).

### Summary measures and planned methods for analysis

For the pooled analysis of PD reduction, CAL gain and bone defect depth reduction, the mean difference (MD) and its standard error (SE) between the two groups were calculated. The presence/absence of heterogeneity was assessed by means of the Higgins Index (*I*^2^). Data were pooled with a fixed-or a random-effect model on the basis of an *I*^2^ lower or upper the cut-off of 50%. The inverse of variance test was used to analyze the overall effects. We combined split-mouth and parallel designs as suggested by Elbourne et al. [16] and we estimated the absence of a carryover effect since we assumed that treated defects were not adjacent [17]. Mean Difference (MD) between test and control and Standard Error (SE) were calculated according to the method described by Lesaffre et al. [18]. In addition, subgroup analysis was performed on the basis of the study design (split-mouth or parallel groups) for PD reduction and CAL gain to investigate systematic differences. A subgroup analysis was performed on the basis of the type of bony defect (intra-bony or furcation defect) for bone defect depth reduction. In addition, subgroup analysis was performed on the basis of the type of bony defects (intra-bony or furcation defects). Trial sequential analysis (TSA) was performed with the goal to assess the power of the meta-analytic findings and to adjust results of the meta-analysis for the presence of types I (5%) and II (10%) errors. In particular, the alpha-spending function, trial sequential monitoring boundaries and the required information size (RIS) were calculated. TSA was performed using a model-variance based approach and performing heterogeneity correction on the basis of meta-analysis results. Results of the TSA were evaluated by graphically assessing if the cumulative Z-curve crossed the trial sequential monitoring boundaries, the futility boundaries and the RIS threshold.

## Results

### Studies selection and studies features

**Fig. 1:**
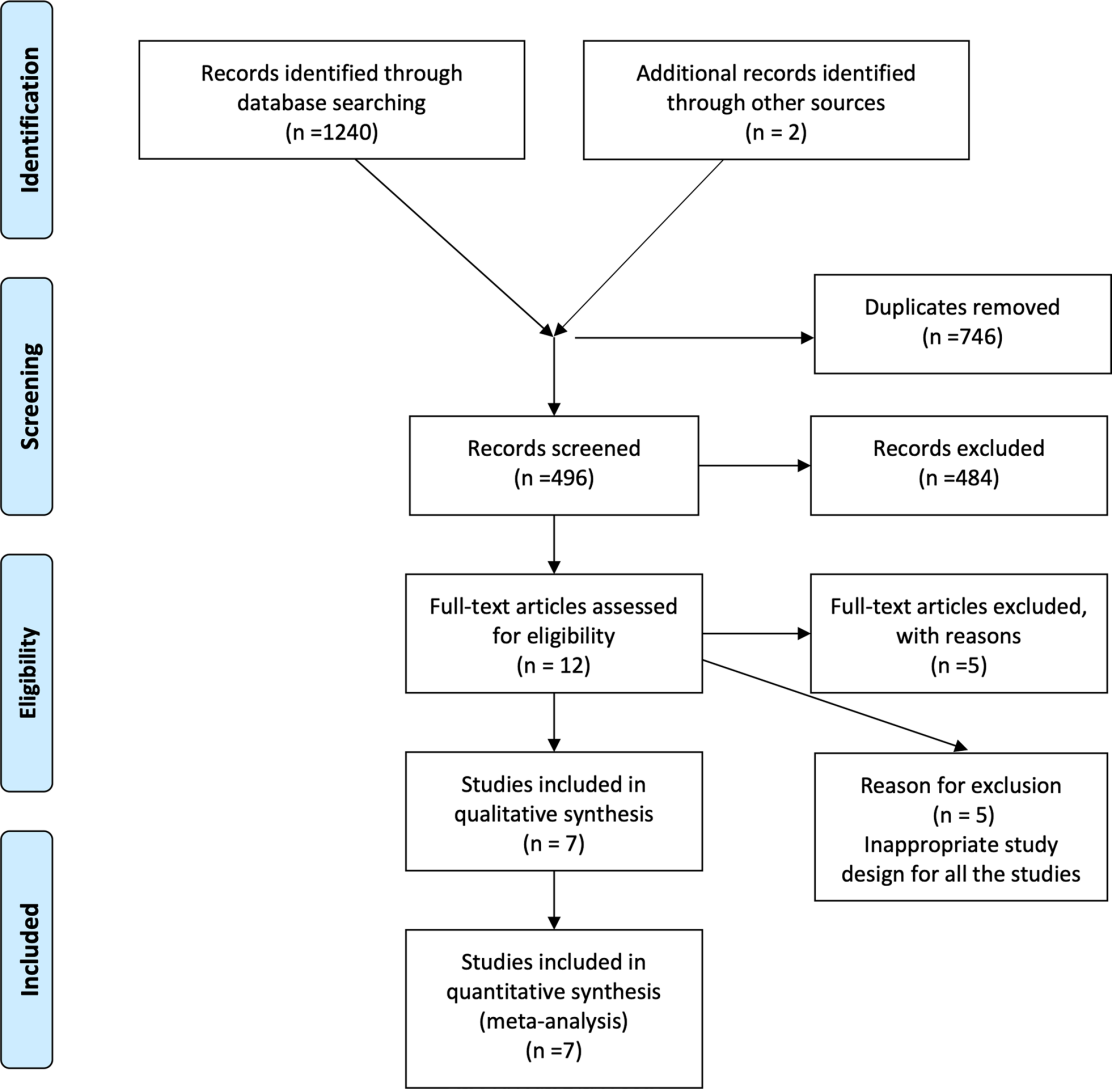
PRISMA flow chart showing different phases of the selection process

A total of 1242 records were screened by title and abstract from electronic databases. After the first round, 12 out of these papers were considered eligible for full-text examination. At the end of full text examination, seven papers met the inclusion criteria and were included in this systematic review [19–25]. The flow chart of the selection process is reported on Fig. 1. All the included studies were RCTs comparing the combination therapy of second step of periodontal therapy + Alendronate gel 1% (test group) with second step of periodontal therapy + placebo (control group). Reasons for exclusion of the remaining paper are reported on Additional file 1. The articles publication years ranged between 2012 and 2018. Two studies reported data about furcation defects [19, 20] while 5 reported data about intra-bony defects [21–25]. All the studies reported a 6-month follow-up [22–25], while only two studies reported a 12-months follow-up [19, 20]. One study had a split mouth design [21], while six had a parallel design [19, 20, 22–25]. Five studies had 2 arms of comparison [20–24], while two had three arms [19, 25]. Six out of seven of the included studies were funded by pharmaceutical companies [19, 20, 22–25]. In all the included studies, alendronate was injected into the periodontal pockets using a syringe with a blunt cannula during step II of periodontal therapy (Table 1).

**Table 1:** Characteristics of the included studies

Authors		Year	Follow-up	Design of the study	Type of defect	Type of intervention		Number of defects		Funding
						Test	Control	Test	Control	
Ipshita et al. [19]	India	2018	6–12 months	Parallel	Inter-radicular	SRP + ALN 1%	SRP + Placebo	30	30	Yes
Dutra et al. [21]	Brasil	2017	6 months	Split-mouth	Intra-bony	ALN 1%	Placebo	20	20	No
Sharma et al. [22]	India	2017	6 months	Parallel	Intra-bony	ALN 1%	Placebo	39	42	Yes
Pradeep et al. [25]	India	2017	6–9 months	Parallel	Intra-bony	ALN 1%	Placebo	30	30	Yes
Sharma and Pradeep [23]	India	2012	6 months	Parallel	Intra-bony	ALN 1%	Placebo	33	33	Yes
Sharma and Pradeep [24]	India	2012	6 months	Parallel	Intra-bony	ALN 1%	Placebo	26	26	Yes
Pradeep et al. [20]	India	2013	6–12 months	Parallel	Inter-radicular	ALN 1%	Placebo	29	28	Yes

**Fig. 2:**
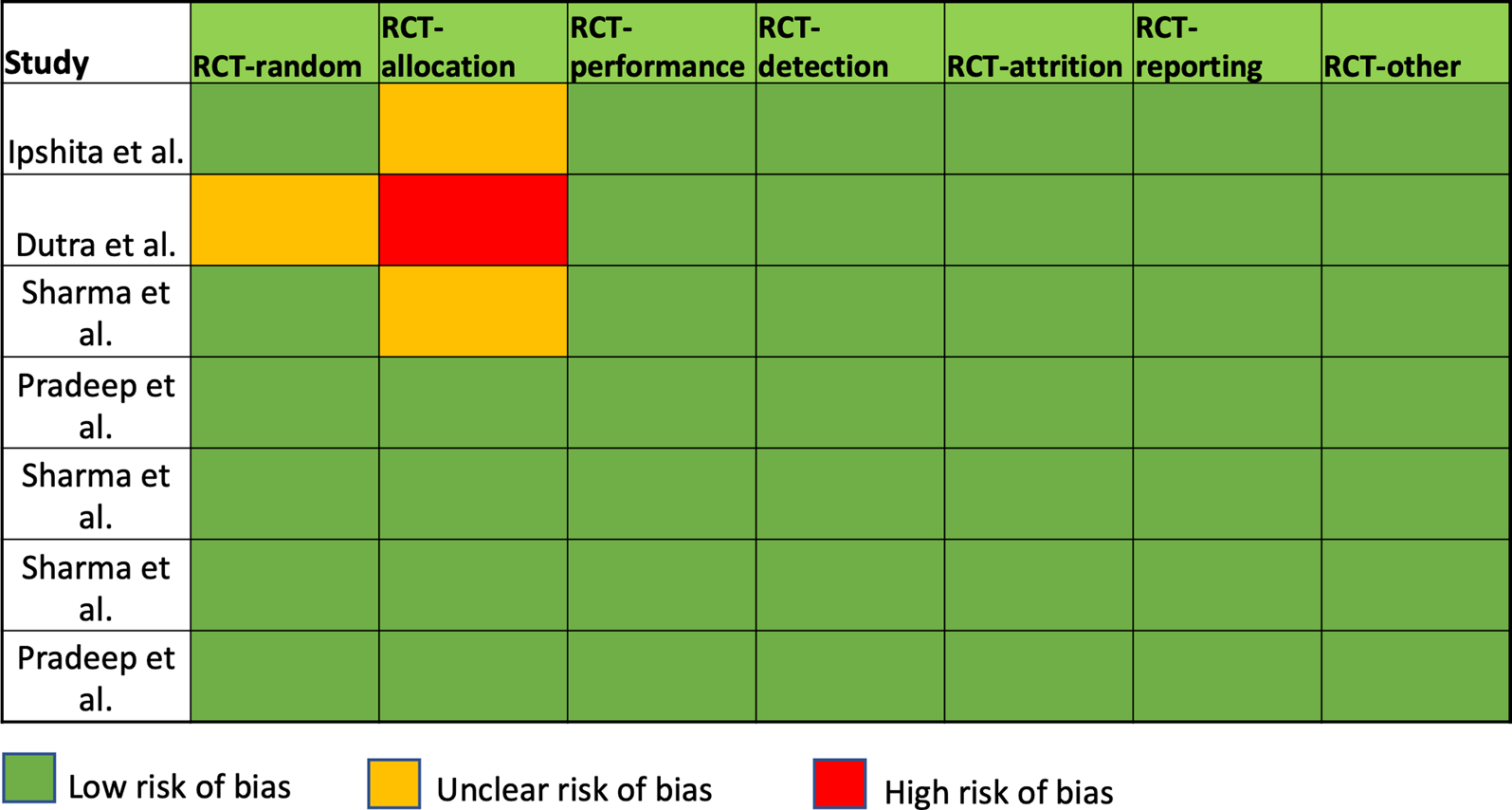
Risk of bias assessment according to the Cochrane Collaboration tool

Outcomes, for which meta-analysis of the included studies was not performed due to lack of data, are resumed in Table 2. Results of the risk of bias assessment are summarized in Fig. 2. One study showed unclear risk of bias for randomization [21] while two studies showed unclear risk of bias for allocation concealment [19, 22] and one study had high risk of bias for allocation concealment [21]. Six studies had low risk of bias [19, 20, 22–25] while one reported high risk of bias [21] (Fig. 2).

**Table 2:** Quantitative synthesis of the included studies

	Parameter	Time interval		Placebo	Alendronate		AV		*p* value
Ipshita et al. [19]	mSBI^a^	Baseline		2.03 ± 0.61	2.10 ± 0.66		2.06 ± 0.73		0.92
		6 months		1.23 ± 0.62	0.70 ± 0.65		0.66 ± 0.60		0.001
		12 months		0.90 ± 0.71	0.50 ± 0.50		0.40 ± 0.49		0.003
	PI	Baseline		2.03 ± 0.40	1.99 ± 0.37		2.01 ± 0.30		0.89
		6 months		0.78 ± 0.10	0.76 ± 0.10		0.77 ± 0.09		0.60
		12 months		0.57 ± 0.09	0.54 ± 0.11		0.55 ± 0.13		0.71
	PD	Baseline		7.06 ± 1.04	7.03 ± 1.12		7.06 ± 1.32		0.99
		6 months		5.53 ± 0.81	4.03 ± 0.55		5.00 ± 0.83		< 0.001
		12 months		5.20 ± 0.66	2.93 ± 0.78		4.63 ± 0.71		< 0.001
	RVCAL (mm)^a^	Baseline		6.23 ± 1.13	6.13 ± 1.00		6.30 ± 0.79		0.80
		6 months		5.06 ± 0.78	3.63 ± 1.10		4.26 ± 0.82		< 0.001
		12 months		4.76 ± 0.72	2.36 ± 0.71		3.76 ± 1.35		< 0.001
	RHCAL (mm)^a^	Baseline		6.06 ± 1.08	5.91 ± 0.92		6.16 ± 0.92		0.56
		6 months		5.03 ± 0.71	3.56 ± 1.07		4.20 ± 1.20		< 0.001
		12 months		4.60 ± 0.62	2.20 ± 0.61		3.93 ± 1.48		< 0.001
	Bone defect depth (mm)^a^	Baseline		5.21 ± 0.33	5.22 ± 0.63		5.49 ± 0.78		0.14
		6 months		5.09 ± 0.37	3.21 ± 0.46		4.73 ± 0.42		< 0.001
		12 months		5.02 ± 0.36	2.86 ± 0.32		4.53 ± 0.90		< 0.001

	Parameters			Baseline		3 months		6 months	
Dutra et al. [21]	PPD (mm)								
	ALN			6.4 ± 1.4		4.3 ± 1.6		4.3 ± 1.6	
	Placebo			6.2 ± 1.6		4.4 ± 1.8		4.4 ± 1.7	
	*P* value			0.468		0.239		0.127	
	CAL (mm)								
	ALN			6.9 ± 1.2		4.3 ± 1.1		3.6 ± 0.7	
	Placebo			6.7 ± 1.1		5.1 ± 0.9		4.3 ± 0.7	
	*p* value			0.356		0.010		0.021	

			Baseline (mm)			6 months (mm)		*p* value	
	CBCT based measurements of bone fill according to the treatment groups over time								
	ALN		4.5 ± 1.8			3.8 ± 2.0		0.003	
	Placebo		5.1 ± 2.2			4.9 ± 2.0		0.099	

	Parameters	Visits		ALN		Placebo		*p* value	
Sharma et al. [22]	Probing depth	Baseline		7.84 ± 2.04		7.62 ± 1.97		0.645	
		2 month		5.05 ± 1.78		6.38 ± 1.84		< 0.002	
		6 month		3.68 ± 1.93		5.57 ± 1.88		< 0.001*	
	Periodontal attachment level	Baseline		6.43 ± 1.77		6.19 ± 1.54		0.531	
		2 month		4.05 ± 1.94		5.22 ± 1.73		< 0.008*	
		6 month		2.49 ± 1.53		4.41 ± 1.83		< 0.001*	
	Intrabony defect depth	Baseline		5.18 ± 1.00		5.10 ± 0.95		0.727	
		6 month		3.07 ± 0.94		4.97 ± 0.95		< 0.001*	

		Baseline		3 months		6 months		12 months	
		ALN	Placebo	ALN	Placebo	ALN	Placebo	ALN	Placebo
Pradeep et al. [20]	Full mouth PI	1.73 ± 0.31	1.80 ± 0.46	0.91 ± 0.27	1.00 ± 0.28	0.20 ± 0.40	0.52 ± 0.24	0.45 ± 0.19	0.55 ± 0.25
	*p* value	0.217		0.251		0.369		0.047*	
	GI (mSBI)	2.54 ± 0.17	2.53 ± 0.16	1.01 ± 0.41	1.15 ± 0.44	0.79 ± 0.12	0.88 ± 0.29	0.85 ± 0.17	0.90 ± 0.21
	*p* value	0.751		0.165		0.059		0.132	

	Parameters	Visit		ALN group		Placebo group		*p* value	
	Clinical and radiographical parameters								
	PD (mm)	Baseline		6.93 ± 0.69		6.77 ± 0.82		0.407	
		3 months		4.43 ± 0.73		5.53 ± 0.73		< 0.001†	
		6 months		3.10 ± 0.71		5.17 ± 0.79		< 0.001	
		12 months		3.14 ± 0.71		5.39 ± 0.74		< 0.001	
	RVCAL	Baseline		7.30 ± 0.79		7.27 ± 0.78		0.846	
		3 months		5.07 ± 0.64		6.33 ± 0.66		< 0.001	
		6 months		4.07 ± 0.64		6.03 ± 0.76		< 0.001	
		12 months		4.07 ± 0.66		6.14 ± 0.89		< 0.001	
	RHCAL	Baseline		8.07 ± 0.64		8.03 ± 0.81		0.994	
		3 months		6.10 ± 0.66		7.17 ± 0.79		< 0.001	
		6 months		5.03 ± 0.56		6.97 ± 0.76		< 0.001	
		12 months		5.00 ± 0.54		7.03 ± 0.79		< 0.001	
	BD depth	Baseline		3.94 ± 0.24		3.94 ± 0.25		0.828	
		6 months		2.67 ± 0.26		3.83 ± 0.25		< 0.001	
		12 months		2.64 ± 0.23		3.84 ± 0.24		< 0.001	

		Visits		ALN	Placebo	T value		*p* value	
Sharma and Pradeep [23]	PD	Baseline		7.85 ± 2.20	7.69 ± 2.22	0.06		0.008	
		2 months		6.04 ± 1.99	6.96 ± 2.10	2.63		0.111	
		6 months		3.96 ± 1.28	6.04 ± 1.68	25.05		< 0.001	
	CAL	Baseline		6.12 ± 1.77	5.96 ± 1.88	0.09		0.763	
		2 months		4.46 ± 2.43	5.19 ± 2.29	1.23		0.271	
		6 months		2.85 ± 1.82	4.54 ± 2.12	9.50		0.003	
	IBD Depth	Baseline		5.45 ± 1.18	5.48 ± 0.92	0.006		0.937	
		6 months		2.95 ± 0.86	5.37 ± 0.90	97.08		< 0.001	
	Mean PD (mm)	2 months		1.81 ± 1.20	0.73 ± 1.58	7.60		0.008	
		6 months		3.88 ± 1.39	1.65 ± 1.35	34.21		< 0.001	
	Mean CAL (mm)	2 months		1.65 ± 1.09	0.77 ± 1.68	5.06		0.029	
		6 months		3.27 ± 1.11	1.42 ± 1.70	21.41		< 0.001	
	Mean IBD depth (mm)	6 months		2.50 ± 0.73	0.10 ± 0.06	273.12		< 0.001	
	Percentage of bone defect fill (%)	6 months		46.1 ± 9.48	2.0 ± 1.20	555.41		< 0.001	

			Alendronate group	Placebo group		t*		*p*	
Sharma and Pradeep [24]	PD	Baseline	7.58 ± 2.13	7.24 ± 2.18		0.39		0.533	
		2 months	4.39 ± 1.45	5.88 ± 1.78		13.74		< 0.014	
		6 months	3.09 ± 1.82	5.09 ± 1.54		23.02		< 0.001	
	CAL	Baseline	6.06 ± 1.82	5.64 ± 1.72		0.95		0.333	
		2 months	3.61 ± 1.99	4.82 ± 1.91		6.33		< 0.001	
		6 months	2.03 ± 1.48	4.03 ± 2.02		20.91		< 0.001	
	IBD depth	Baseline	4.70 ± 1.00	4.71 ± 1.04		0.01		0.971	
		6 months	2.82 ± 0.87	4.60 ± 1.06		5.43		< 0.001	

			Alendronate group	Placebo group		t*		*p*	
	Change in PD, CAL, IBD, and Bone Fill in ALN and Placebo Groups from baseline to 2 and 6 months								
	PD (mm; mean—SD)	2 months	3.18 ± 1.28	1.36 ± 0.85		45.60		< 0.001	
		6 months	4.48 ± 1.27	2.15 ± 1.12		62.16		< 0.001	
	CAL (mm; mean–SD)	2 months	2.45 ± 0.75	0.82 ± 0.58		97.20		< 0.001	
		6 months	4.03 ± 0.84	1.61 ± 0.86		132.47		< 0.001	
	IBD depth (mm; mean–SD)	6 months	1.88 ± 0.58	0.10 ± 0.03		304.86		< 0.001	
	Bone-defect fill (%; mean–SD)	6 months	40.4 ± 11.71	2.5 ± 1.02		344.45		< 0.001	

		Placebo		ATV		ALN		*p* value	
Pradeep et al. [25]	Probing depth, clinical attachment level, and intrabony defect depth in placebo, ATV, and ALN at different time intervals								
	PD	Baseline	6.76 ± 1.22	6.56 ± 1.38		6.96 ± 1.12		0–466	
		3 months	6.43 ± 1.22	5.50 ± 1.07		5.43 ± 0.89		< 0.0001*	
		6 months	5.70 ± 0.87	4.10 ± 0.84		3.40 ± 0.49		< 0.0001*	
		9 months	5.26 ± 0.63	3.03 ± 0.71		2.66 ± 054		< 0.0001*	
	CAL	Baseline	6.13 ± 1.13	6.13 ± 0.89		5.93 ± 0.94		0.671	
		3 months	5.96 ± 1.18	4.46 ± 0.73		4.86 ± 0.81		< 0.0001	
		6 months	4.96 ± 0.71	2.43 ± 0.50		3.6 ± 1.10		< 0.0001	
		9 months	4.63 ± 0.61	1.86 ± 0.43		2.43 ± 0.77		< 0.0001	
	IBD depth	Baseline	5.17 ± 0.35	5.46 ± 0.81		5.18 ± 0.65		0.145	
		6 months	5.07 ± 0.38	3.56 ± 0.54		3.05 ± 0.36		< 0.0001	
		9 months	5.04 ± 0.38	3.47 ± 0.70		2.79 ± 0.30		< 0.0001	

			Placebo	ATV		ALN		*p* value	
	PD	Baseline-3 months	0.33 ± 0.47	1.06 ± 0.63		1.53 ± 0.50		< 0.001	
		Baseline-6 months	1.06 ± 0.90	2.46 ± 0.97		3.56 ± 1.13		< 0.001	
		Baseline-9 months	1.50 ± 1.07	3.53 ± 1.27		4.30 ± 1.39		< 0.001	
	CAL	Baseline-3 months	0.16 ± 0.37	1.66 ± 0.60		1.06 ± 0.52		< 0.001	
		Baseline-6 months	1.16 ± 0.94	3.7 ± 0.91		2.33 ± 1.34		< 0.001	
		Baseline-9 months	1.50 ± 1.07	4.26 ± 1.08		3.50 ± 1.16		< 0.001	
	IBD depth	Baseline-6 months	0.09 ± 0.17	1.90 ± 0.44		2.12 ± 0.34		< 0.001	
		Baseline-9 months	0.12 ± 0.17	1.98 ± 0.72		2.38 ± 0.45		< 0.001	
	Radiographic DDR	Baseline-6 months	1.86 ± 3.56	34.61 ± 5.11		40.87 ± 2.73		< 0.001	
		Baseline-9 months	2.44 ± 3.48	35.92 ± 10.04		45.82 ± 3.83		< 0.001

### Meta-analysis and trial sequential analysis

A total number of 408 patients were treated in the included studies in both test and control groups. A total of 207 sites were treated in the test group while 209 sites were treated in the control group.

#### PD reduction

**Fig. 3:**
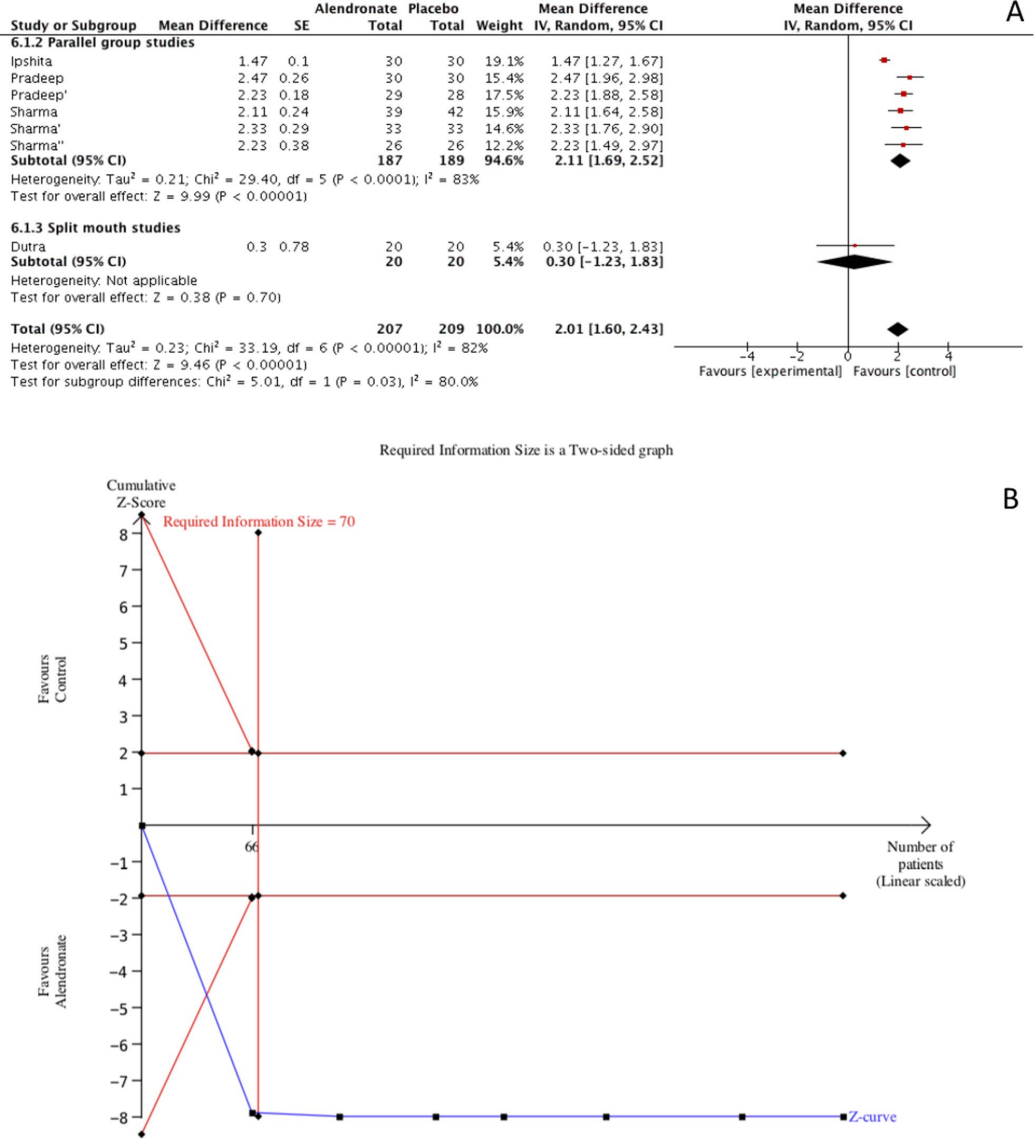
Meta-analysis (**A**) and Trial sequential analysis (**B**) for the effects of Alendronate therapy on PD reduction

Meta-analysis of PD reduction showed a significant difference (*p* < 0.00001) when alendronate was topically applied during step II periodontal therapy compared to placebo; MD = 2.01 (95% CI [1.60, 2.43]). Such results were characterized by a high rate of heterogeneity (I^2^ = 82%), for such reason a random effects model was used for data interpolation. A subgroup analysis was performed based on study design (parallel-groups vs split-mouth) to test for a possible influence of study design on the analyzed outcome. The effect estimate for parallel group studies was 2.11 (95% CI [1.69, 2.51]) and for split-mouth 0.30 (95% CI [− 1.23, 1.83]) and although both groups showed a benefit for alendronate compared to placebo an important difference was detected. Results of the TSA confirmed the previously performed meta-analysis with the z-curve crossing the lower alpha spending boundary, in addition the meta-analysis was characterized by a good power of evidence since the RIS (74 patients) was crossed (Fig. 3).

#### CAL gain

**Fig. 4:**
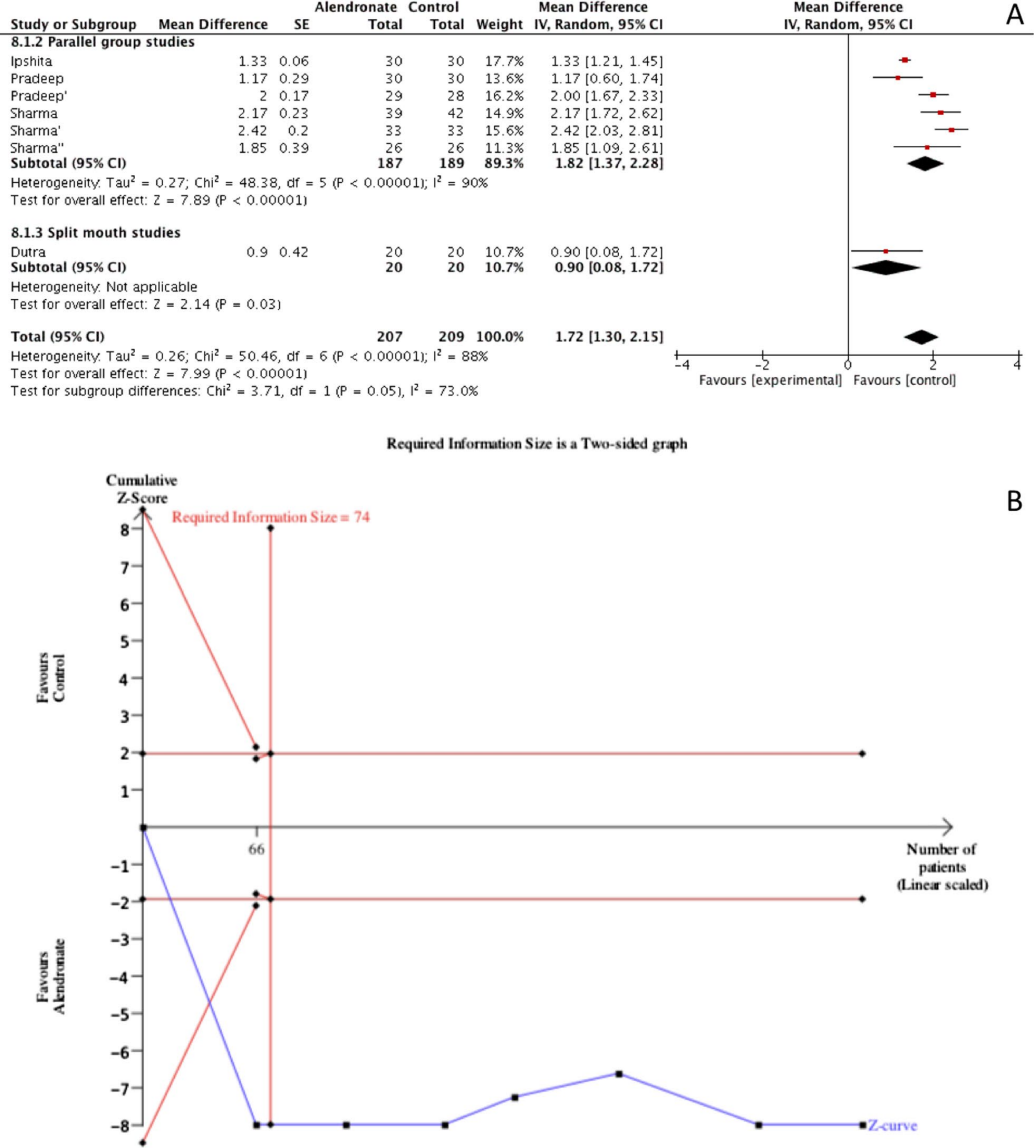
Meta-analysis (**A**) and Trial sequential analysis (**B**) for the effects of Alendronate therapy on CAL gain

Meta-analysis of CAL gain revealed a significant improvement (*p* < 0.00001) when alendronate was topically applied, MD = 1.72 (95% CI [1.30, 2.15]). Such results were characterized by a high rate of heterogeneity among studies (*I*^2^ = 88%) and for this reason a random effect model was used for data interpolation. A sub-group analysis was performed based on study design (parallel groups vs split-mouth); the effect estimate for parallel group studies was 1.82 (95% CI [1.37, 2.28]) and for split-mouth 0.90 (95% CI [0.08, 1.72]), also in this case study design influenced the effect size of results. The TSA confirmed the previously performed meta-analysis with the z-curve crossing the lower alpha spending boundary, in addition the meta-analysis was characterized by a good power of evidence since the RIS (74 patients) was also crossed (Fig. 4).

#### Bone defect depth reduction

**Fig. 5:**
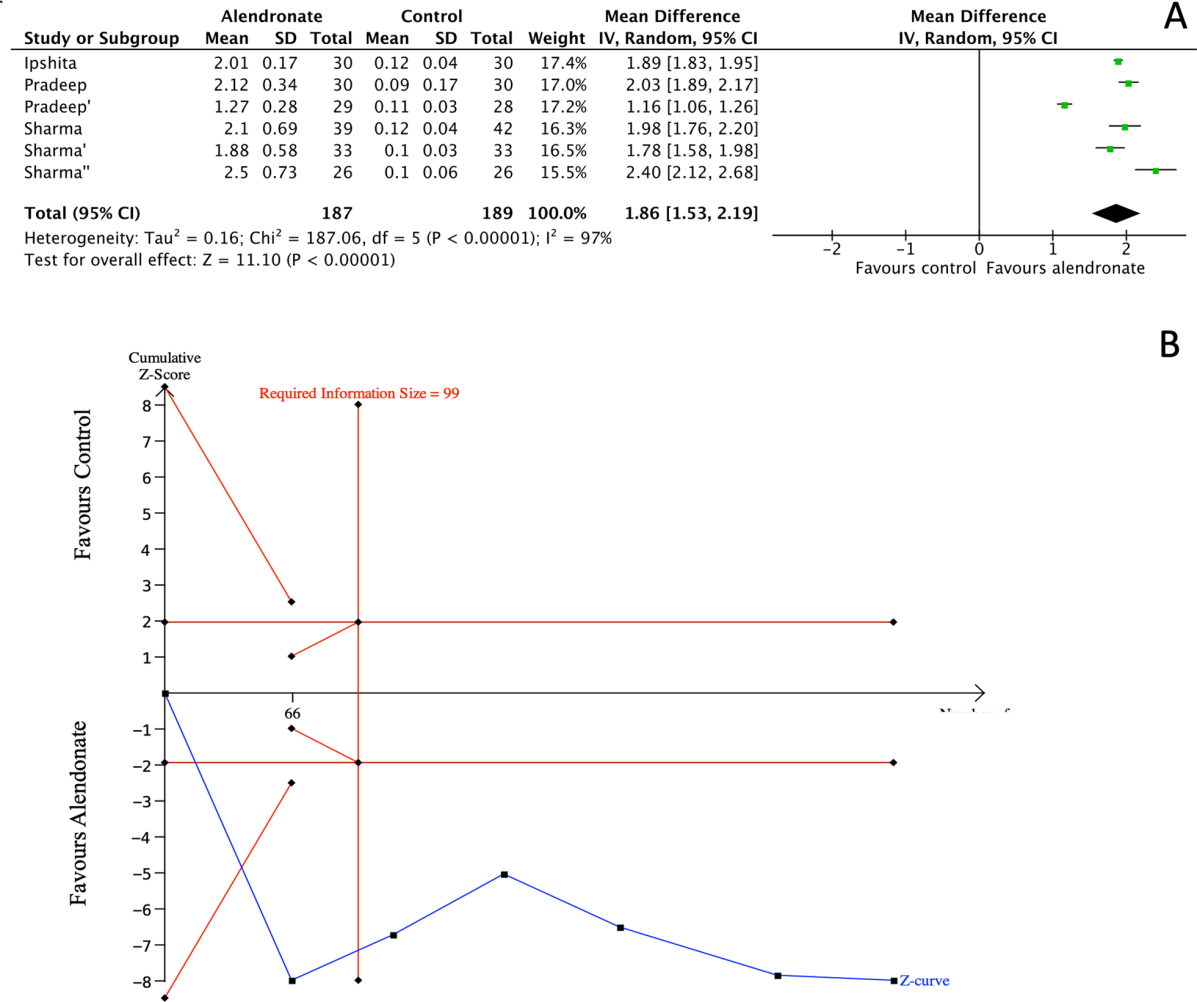
Meta-analysis (**A**) and Trial sequential analysis (**B**) for the effects of Alendronate therapy on bone defect depth reduction

The analysis of bone defect depth reduction shows a significant difference between the therapy with alendronate compared to control: MD = 1.86 (95% CI [1.53, 2.19]), with results characterized by a high rate of heterogeneity (*I*^2^ = 97%). TSA confirmed such findings with the z-curve crossing the lower alpha spending boundary and RIS threshold (99 patients). No differences were detected between intra-bony and inter-radicular defects (*p* = 0.18) (Fig. 5).

### GRADE

Evidence by subgroups was qualified using the GRADE approach. Low quality of evidence supports the beneficial effect of alendronate 1% as an adjunct to second step of periodontal therapy in the treatment of intra-bony and inter-radicular defects. The level of evidence was downgraded due to inconsistency results of the included studies. Results of the GRADE evaluation are summarized in Additional file 1.

## Discussion

This systematic review aimed to provide a clear focus on the role of alendronate 1% as an adjunct to second step of periodontal therapy in treatment of intra-bony and inter-radicular defects. Since the introduction of host-modulators in the treatment of periodontal disease [26], several studies have been performed to analyze the role of different substances in combination with second step of periodontal therapy [27–31].

Results of the present study showed that the topical application of Alendronate gel 1% can provide a significant benefit in PD reduction, CAL gain and bone defect depth reduction when applied after non-surgical periodontal treatment. A significant difference between parallel groups and split-mouth design was detected when subgroup analysis was performed for PD reduction and CAL gain. No significant differences were detected between intra-bony and inter-radicular defects when subgroup analysis was performed for bone defect depth reduction. BPs are often administered orally for the treatment of post-menopausal osteoporosis showing a wide range of adverse events including gastro-intestinal side effects, acute phase syndrome, secondary hyperparathyroidism, hypocalcemia, musculoskeletal pain and osteonecrosis of the jaw; therefore local application of alendronate might appear to be safer and devoid of side effects since none of the studies had drop-out patients that seemed to be related to local treatment; furthermore local administration offers the advantage of reaching a higher concentration of drug at the osseous defect with a reduced dosage [32]. However, even if these results might seem promising in providing a beneficial effect as an adjunct to subgingival instrumentation of intra-bony and inter-radicular defects, there are several concerns that must be taken into account when interpreting these results.

First of all, only two of the included studies [19, 20] reported data about inter-radicular defects, hence further studies are needed to confirm the results obtained of a potential beneficial effect of alendronate in this subtype of defects.

Of the seven included studies, only one provided more detailed information about the subgingival instrumentations performed [21]; in the remaining six it was not considered the number of sessions performed, the kind of instruments that were used or the experience of the operators who had performed the treatment. Moreover, the adjunct of alendronate during subgingival instrumentation is an adjunctive cost to conventional step II of periodontitis treatment.

We did not set the smoking status as an exclusion criterion, so this factor should be considered in the outcome of treatment, even if the use of alendronate as a host modulator in intraosseous defects could be particularly important for smokers whose healing capacity is impaired [33, 34]. Since only two studies [19, 20] had a 12-months follow-up, studies with a longer follow-up are necessary to confirm such results. An important point that should be considered when interpreting these results is that six studies [19, 20, 22–25] out of seven were conducted in the same country (India). Therefore, it must be considered the risk of a geographical bias, data from a more heterogeneous population would play an important role in confirming these findings. In addition, six [19, 20, 22–25] out of seven studies were funded by external pharmaceutical companies providing a potential confounding effect in the interpretation of results. An important issue is that one of the included studies [21] had a split-mouth design. Since its introduction by Ramfjord et al. in 1968 [35], split-mouth design has been widely used in oral health related studies. It generally requires less patients since the same individual serves as both test and control. However this kind of design presents some critical aspects as carry-across effect and needs a more complicated analysis in comparison with whole mouth studies [17, 36]. These results are consistent with the findings of Donos et al. [37] and the EFP Stage I-III periodontitis guideline [3] which affirm that only the adjunct of antiseptics and antibiotics may provide a beneficial effects to step II of periodontal therapy.

## Conclusion

Based on the results available from these RCTs, this review shows that local delivery of alendronate seems to be effective in improving PD, CAL and bone defect depth. However, studies conducted by different research groups and on a geographically more heterogeneous population with a more standardized protocol are necessary to confirm these findings. Therefore, even if topical administration of Alendronate could have a potential beneficial effect on periodontitis clinical parameters, multicentric studies with a longer follow-up are needed to clarify this point.

## Supplementary Information

**Additional file 1.** Results of the GRADE evaluation.

## Data Availability

Not applicable.

## Abbreviations

CAL: Clinical attachment level
MD: Mean difference
PD: Probing depth
RCT: Randomized clinical trial
SE: Standard error
TSA: Trial sequential analysis
