# Supplementary material for: Added effect of 1% topical alendronate in intra-bony and inter-radicular defects as part of step II periodontal therapy: a systematic review with meta-analysis and trial sequential analysis
Source: BMC Oral Health. 2022 Jan 21;22:15. doi: 10.1186/s12903-022-02044-1 (PMC8780760; doi:10.1186/s12903-022-02044-1)
Supplement: Supplementary file 1 — Additional file 1. Results of the GRADE evaluation. [file 12903_2022_2044_MOESM1_ESM.docx]

**Adjunct effect of topical alendronate therapy for the non-surgical treatment of periodontal defects: systematic review, meta-analysis and trial sequential analysis**

Claudia Arena^1^, Vito Carlo Alberto Caponio^1^, Khrystyna Zhurakivska^1^, Lucio Lo Russo ^1^, Lorenzo Lo Muzio^1^, Giuseppe Troiano^1^.

1. Department of Clinical and Experimental Medicine, University of Foggia, Foggia, Italy.

**Corresponding author:**

Giuseppe Troiano, DDS, PhD

Department of Clinical and Experimental Medicine,

University of Foggia, Foggia 71122, Italy , Ph: +390881588082

email: giuseppe.troiano@unifg.it

**Supplementary material legend**

**Appendix 1**: Meta-analysis for the effects of Alendronate therapy on PD reduction by sub-groups.

**Appendix 2**: Meta-analysis for the effects of Alendronate therapy on CAL gain by sub-groups.

**Appendix 3**: Meta-analysis for the effects of Alendronate therapy on bone defect depth reduction by sub-groups.

**Appendix 4:** Reasons for exclusion of articles read in full-text.

**Appendix 5**: GRADE for the effects of Alendronate therapy on PD reduction.

**Appendix 6**: GRADE for the effects of Alendronate therapy on CAL gain.

**Appendix 7**: GRADE for the effects of Alendronate therapy on bone defect depth reduction.

**Appendix 1**

**Appendix 2**

**Appendix 3**

**Appendix 4**

**
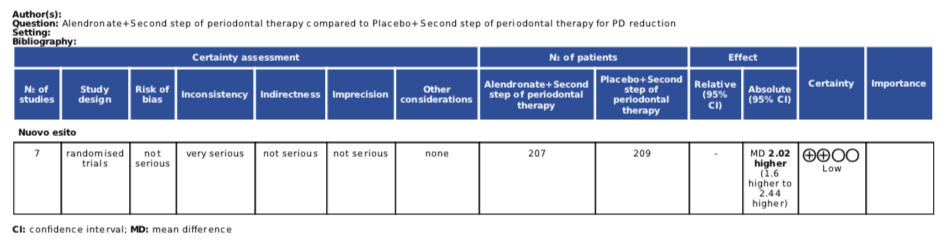
**

**Appendix 5**

**
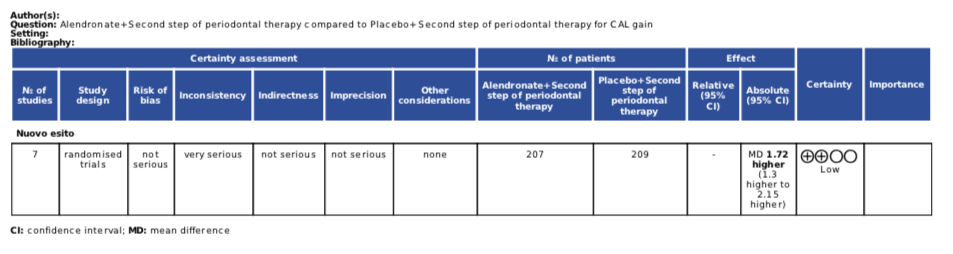
**

**Appendix 6**

**
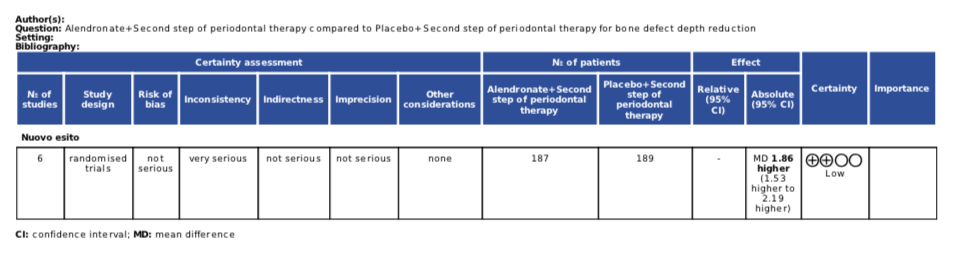
**

**Sharma:** Sharma A, Raman A, Pradeep AR (2017) Role of 1% alendronate gel as adjunct to mechanical therapy in the treatment of chronic periodontitis among smokers. J Appl Oral Sci 25 (3):243-249. doi:10.1590/1678-7757-2016-0201

**Sharma’:** Clinical efficacy of 1% alendronate gel as a local drug delivery system in the treatment of chronic periodontitis: a randomized, controlled clinical trial. J Periodontol 83 (1):11-18. doi:10.1902/jop.2011.110091

**Sharma’’:** Sharma A, Pradeep AR (2012) Clinical efficacy of 1% alendronate gel in adjunct to mechanotherapy in the treatment of aggressive periodontitis: a randomized controlled clinical trial. J Periodontol 83 (1):19-26. doi:10.1902/jop.2011.110206

**Pradeep:** Pradeep AR, Kanoriya D, Singhal S, Garg V, Manohar B, Chatterjee A (2017) Comparative evaluation of subgingivally delivered 1% alendronate versus 1.2% atorvastatin gel in treatment of chronic periodontitis: a randomized placebo-controlled clinical trial. J Investig Clin Dent 8 (3). doi:10.1111/jicd.12215

**Pradeep’:** Pradeep AR, Kumari M, Rao NS, Naik SB (2013) 1% alendronate gel as local drug delivery in the treatment of Class II furcation defects: a randomized controlled clinical trial. J Periodontol 84 (3):307-315. doi:10.1902/jop.2012.110729
